# Supplementary material for: Multi‐dataset identification of innovative feature genes and molecular mechanisms in keratoconus
Source: J Cell Mol Med. 2024 Sep 19;28(18):e70079. doi: 10.1111/jcmm.70079 (PMC11412914; doi:10.1111/jcmm.70079)
Supplement: Supplementary file 2 — Material S2. [file JCMM-28-e70079-s002.zip › 7.GSEA analysis/5.RNF217-AS1/KEGG_MTOR_SIGNALING_PATHWAY.html]

Details for gene set KEGG\_MTOR\_SIGNALING\_PATHWAY[GSEA]

|  || Dataset | RNF217-AS1.group.cls#h\_versus\_l |
| Phenotype | group.cls#h\_versus\_l |
| Upregulated in class | h |
| GeneSet | KEGG\_MTOR\_SIGNALING\_PATHWAY |
| Enrichment Score (ES) | 0.19128704 |
| Normalized Enrichment Score (NES) | 0.65415365 |
| Nominal p-value | 0.8492063 |
| FDR q-value | 0.9601642 |
| FWER p-Value | 1.0 |
Table: GSEA Results Summary

  

Fig 1: Enrichment plot: KEGG\_MTOR\_SIGNALING\_PATHWAY      
 Profile of the Running ES Score & Positions of GeneSet Members on the Rank Ordered List

  

| SYMBOL | TITLE | RANK IN GENE LIST | RANK METRIC SCORE | RUNNING ES | CORE ENRICHMENT || 1 | PIK3R5 | na | 1058 | 0.434 | 0.0694 | Yes |
| 2 | TSC2 | na | 4977 | 0.218 | 0.0064 | Yes |
| 3 | BRAF | na | 5517 | 0.199 | 0.0366 | Yes |
| 4 | CAB39 | na | 5654 | 0.194 | 0.0773 | Yes |
| 5 | VEGFA | na | 5789 | 0.190 | 0.1171 | Yes |
| 6 | TSC1 | na | 6131 | 0.179 | 0.1485 | Yes |
| 7 | PIK3CD | na | 6778 | 0.159 | 0.1664 | Yes |
| 8 | RPS6KA2 | na | 7112 | 0.150 | 0.1913 | Yes |
| 9 | PIK3CB | na | 11439 | 0.064 | 0.0812 | No |
| 10 | RPS6 | na | 11627 | 0.061 | 0.0898 | No |
| 11 | MTOR | na | 11752 | 0.059 | 0.0997 | No |
| 12 | PDPK1 | na | 12622 | 0.047 | 0.0854 | No |
| 13 | IGF1 | na | 13325 | 0.038 | 0.0740 | No |
| 14 | MAPK1 | na | 13362 | 0.038 | 0.0817 | No |
| 15 | EIF4B | na | 13560 | 0.035 | 0.0841 | No |
| 16 | STK11 | na | 13595 | 0.035 | 0.0912 | No |
| 17 | PIK3CA | na | 14021 | 0.031 | 0.0860 | No |
| 18 | MAPK3 | na | 14127 | 0.029 | 0.0897 | No |
| 19 | RPS6KA3 | na | 14828 | 0.021 | 0.0743 | No |
| 20 | ULK1 | na | 15064 | 0.018 | 0.0717 | No |
| 21 | RICTOR | na | 15236 | 0.017 | 0.0706 | No |
| 22 | EIF4E | na | 15571 | 0.013 | 0.0641 | No |
| 23 | AKT2 | na | 15816 | 0.011 | 0.0595 | No |
| 24 | PIK3R1 | na | 16145 | 0.007 | 0.0517 | No |
| 25 | RPS6KB2 | na | 16186 | 0.007 | 0.0521 | No |
| 26 | RPTOR | na | 17040 | -0.002 | 0.0280 | No |
| 27 | RPS6KB1 | na | 17704 | -0.008 | 0.0107 | No |
| 28 | ULK2 | na | 18031 | -0.011 | 0.0039 | No |
| 29 | DDIT4 | na | 18070 | -0.011 | 0.0054 | No |
| 30 | AKT1 | na | 18280 | -0.013 | 0.0024 | No |
| 31 | EIF4EBP1 | na | 18305 | -0.014 | 0.0049 | No |
| 32 | RPS6KA1 | na | 18901 | -0.019 | -0.0079 | No |
| 33 | PGF | na | 19309 | -0.023 | -0.0143 | No |
| 34 | VEGFB | na | 19507 | -0.025 | -0.0141 | No |
| 35 | INS | na | 20462 | -0.035 | -0.0337 | No |
| 36 | HIF1A | na | 20692 | -0.037 | -0.0318 | No |
| 37 | EIF4E2 | na | 21700 | -0.047 | -0.0500 | No |
| 38 | CAB39L | na | 22069 | -0.052 | -0.0488 | No |
| 39 | EIF4E1B | na | 22361 | -0.055 | -0.0446 | No |
| 40 | PIK3R2 | na | 22894 | -0.061 | -0.0460 | No |
| 41 | ULK3 | na | 23542 | -0.069 | -0.0488 | No |
| 42 | PRKAA1 | na | 23950 | -0.074 | -0.0435 | No |
| 43 | RHEB | na | 23988 | -0.075 | -0.0273 | No |
| 44 | MLST8 | na | 25083 | -0.092 | -0.0378 | No |
| 45 | STRADA | na | 25341 | -0.096 | -0.0231 | No |
| 46 | VEGFC | na | 26389 | -0.114 | -0.0271 | No |
| 47 | RPS6KA6 | na | 27330 | -0.133 | -0.0237 | No |
| 48 | PRKAA2 | na | 29109 | -0.179 | -0.0337 | No |
| 49 | PIK3CG | na | 30335 | -0.218 | -0.0191 | No |
| 50 | AKT3 | na | 31832 | -0.281 | 0.0025 | No |
| 51 | PIK3R3 | na | 32895 | -0.352 | 0.0529 | No |
Table: GSEA details [plain text format]

  

Fig 2: KEGG\_MTOR\_SIGNALING\_PATHWAY      
 Blue-Pink O' Gram in the Space of the Analyzed GeneSet

  

Fig 3: KEGG\_MTOR\_SIGNALING\_PATHWAY: Random ES distribution      
 Gene set null distribution of ES for **KEGG\_MTOR\_SIGNALING\_PATHWAY**
